# Comparative Evaluation of Microleakage With Total-Etch, Universal (Self-Etch Mode), and Nano Adhesive Systems in Class V Composite Restorations: An In-Vitro Study

**DOI:** 10.7759/cureus.46766

**Published:** 2023-10-09

**Authors:** Sarveshwari Singh, Upendra S Bhadauria, Apoorva Sharma, Rouble Verma Mathur

**Affiliations:** 1 Conservative Dentistry and Endodontics, Sardar Patel Post Graduate Institute of Dental and Medical Sciences, Lucknow, IND; 2 Public Health Dentistry, All India Institute of Medical Sciences, New Delhi, IND; 3 Oral and Maxillofacial Pathology, Sardar Patel Post Graduate Institute of Dental and Medical Sciences, Lucknow, IND; 4 Oral Medicine and Radiology, College of Dental Science, Rajasthan University of Health Sciences, Jaipur, IND

**Keywords:** nano adhesives, composite, microleakage, bonding agents, adhesive system

## Abstract

Objectives: The adhesion of bonding agents and their longevity are of interest to dentistry. Microleakage remains the major cause of composite restoration failures, which in turn depends on bonding between the restorative material and tooth substrate. The objective of this study is to evaluate and compare the microleakage with total-etch, universal, and nano adhesive systems in Class V composite restorations, utilizing a dye penetration method.

Methods: Forty-five extracted premolars were included in the present study, and a Class V cavity on the facial surface of each tooth was prepared. The samples were divided into three groups of 15 teeth each depending on the bonding agents used, following which composite restoration was done. Specimens were thermocycled, and nail varnish was applied except around the restorations. Specimens were then immersed in 2% methylene blue for 24 hours and rinsed; sectioning was done and viewed under a stereomicroscope with 10X magnification.

Results: The mean microleakage score was reported to be significantly higher in the universal adhesive system in the self-etch mode (3.60 ± 0.55) when compared with the total-etch adhesive system (2.40 ± 0.55) and least in the nano adhesive system (1.20+.45) (p value <.05).

Conclusion: The study findings revealed that nano adhesives showed lesser microleakage as compared to total-etch and universal adhesive systems.

Clinical significance: The study strengthens the findings that the nano adhesives have reduced microleakage, resulting in better marginal integrity and increased longevity of restoration. This study signifies that an eighth-generation bonding agent is reported to be better than the other bonding agents used in this study.

## Introduction

The field of adhesive dentistry has undergone great transformation and progress in the last few decades [1]. The introduction of bonding systems in adhesive dentistry aided in the retention and stabilisation of a tooth-coloured restoration. The penetration of adhesive into the demineralized intratubular and intertubular dentin and the formation of resin tags are effective interlocking mechanisms that can seal and prevent permeability through dentin [2]. Even the concept of extension for prevention has changed to the prevention of extension from the dental surgeon's perspective [3].

One of the best predictors of the long-term success of bonded restorations is the ability of marginal sealing [4]. The passage of oral fluids, bacteria, molecules, and ions between cavity walls and restorative material is termed microleakage [4]. Microleakage has also been linked to many postoperative failures, such as sensitivity, recurrent caries, pulpal damage, and fracture of the restorative material. Hence, an important consideration when applying dental adhesive is preventing microleakage along with strong bonding between the cavity wall and restorative material [5].

With the rapid change in technologies, the adhesive systems have also evolved from no-etch to total-etch (fourth and fifth generations) [6]. These total-etch systems (fifth-generation bonding agents) used today combine the primer and adhesive in a single bottle, but a prebonding step of total acid etching still needs to be done [7]. Self-etch adhesives may be two steps or one step. A two-step self-etch adhesive system (sixth-generation bonding agent) consists of acidic primer and bonding resin separately and is sought to eliminate the etching step [1]. On the other hand, a one-step self-etch adhesive system (seventh-generation bonding agent) was further introduced to simplify the bonding protocol. It contains a combination of etchant, primer, and bonding agent in one (all-in-one system) component and is applied in a single step [8].

More recently, a new family of dentin adhesives has been introduced: universal or multi-mode adhesives, which may be used either as etch-and-rinse or as self-etch adhesives [6]. Development in nanotechnology has led to the development of nano adhesives (eighth-generation bonding agents), which contain nanosized crosslinking silica particles. In the new agents, the addition of nanofillers (SiO2) with an average particle size of 12 nm increases the penetration of resin monomers and the hybrid layer thickness, which in turn improves the mechanical properties of bonding systems [6].

Though several studies have been conducted to evaluate the microleakage of the bonding agents of different generations [3,4], very few studies have been conducted to evaluate the microleakage of total-etch, universal, and nano adhesive systems. This present study was, thus, carried out to investigate and compare the microleakage of composite resin with total-etch, universal, and nano adhesive systems in Class V restorations using a stereomicroscope.

## Materials and methods

The present in vitro study was carried out in the Department of Conservative Dentistry and Endodontics, Sardar Patel Post Graduate Institute of Dental and Medical Sciences, Lucknow, India, in collaboration with Sanjay Gandhi Post Graduate Institute of Medical Sciences, Lucknow, India, and Birbal Sahni Institute of Palaeosciences, Lucknow, India. The ethical permission to carry out the study was obtained from the institutional review board of the Sardar Patel Post Graduate Institute of Dental and Medical Sciences, Lucknow.

Armamentarium

Airotor handpiece (NSK Pana Air, Japan), round bur (S.S White, Germany), straight fissure diamond bur (S.S White, Germany), ultrasonic scaler (Woodpecker, China), autoclave (Sun Technologies, India), pumice powder, plaster of Paris, 37% phosphoric acid (Ivoclar, Europe), Tetric N-Bond (Ivoclar, Europe), Prime & Bond universal (Dentsply, Switzerland), Futurabond DC (Voco, Germany), Ceram X One composite (Dentsply, Switzerland), composite handling instrument (GDC, India), LED light curing unit (Elipar, 3M, USA), finishing and polishing disks (3M, USA), contra-angle handpiece (NSK, Japan), periodontal probe (Hu Friedy, USA), distilled water (Nirma Ltd., Sachana, India), nail varnish (Loreal), green stick compound (GC India), 2% methylene blue dye (CDH Lab, India), micromotor (Marathon, India), straight handpiece (NSK, Japan), diamond disc (Horico, Germany), mandrill, and stereomicroscope (Techno Scientific Instruments, Leica).

Eligibility criteria

The inclusion criteria comprised fully developed maxillary and mandibular premolars, non-restored teeth, and teeth without external or internal resorption. Grossly decayed or fractured teeth or teeth with developmental anomalies were excluded from the study.

Sample size estimation

A pilot study was conducted on 15 samples, divided into three groups of five samples each, to determine the feasibility and applicability of the study, as well as the sample size. The sample size estimation was carried out using G*Power software by obtaining the meaningful difference between the three groups, and the estimated sample size was found to be 45. The present study, thus, consisted of 45 extracted maxillary and mandibular premolars divided into three groups.

Sample preparation and handling

The extracted premolars were cleaned under running water, and the stains and calculus were removed by ultrasonic scaling and polished with pumice powder. The selected teeth were autoclaved at 121 degrees Celsius for 15 minutes and then stored in distilled water until further experimentation.

All the selected premolars were mounted on plaster of Paris blocks. A Class V cavity was prepared on the facial surface of each tooth, of dimensions approximately 3 mm wide mesio-distally, 1.5 mm deep, and 2.5 mm high occluso-cervically, parallel to the cemento-enamel junction, using a high-speed airotor handpiece, a round bur, and a straight fissure diamond bur under continuous water spray coolant. The teeth were then randomly divided into three groups: Group A, total-etch (Tetric N-Bond, Ivoclar); Group B, self-etch universal (Prime & Bond Universal, Dentsply); and Group C, nano adhesive (Futura Bond DC, Voco) of 15 samples each, on the basis of the adhesive system used. The allocation of the sample was carried out by a person independent of the experiment.

In Group A (total-etch), the etchant was applied with a syringe, and after waiting for 15 seconds, it was rinsed with water and dried. A single coat of bonding agent was applied with the applicator tip, brushed on dentin for at least 10 seconds, and then light-cured for 20 seconds. Group B (self-etch universal) consisted of the application of adhesive with a microbrush, followed by gentle air dispersion and light curing for 20 seconds. In Group C (nano adhesive), the two liquids were mixed, and then the adhesive was applied with a microbrush, followed by gentle air dispersion and light curing for 20 seconds.

Subsequently, the application of Ceram X One composite with composite handling instruments in increments was done, and then each increment was light-cured for 40 seconds. After restoration of all the samples, finishing and polishing were done using abrasive disks. All the samples were then thermocycled using a thermocycler for 200 cycles at 5- and 55-degree C to understand cyclic exposure to hot and cold temperatures with a dwell time of 30 seconds. Nail varnish was applied over the samples, except around the restoration margins, and a green stick compound was used to seal the apices completely. The samples were then immersed in 2% methylene blue dye for 24 hours.

**Figure 1:**
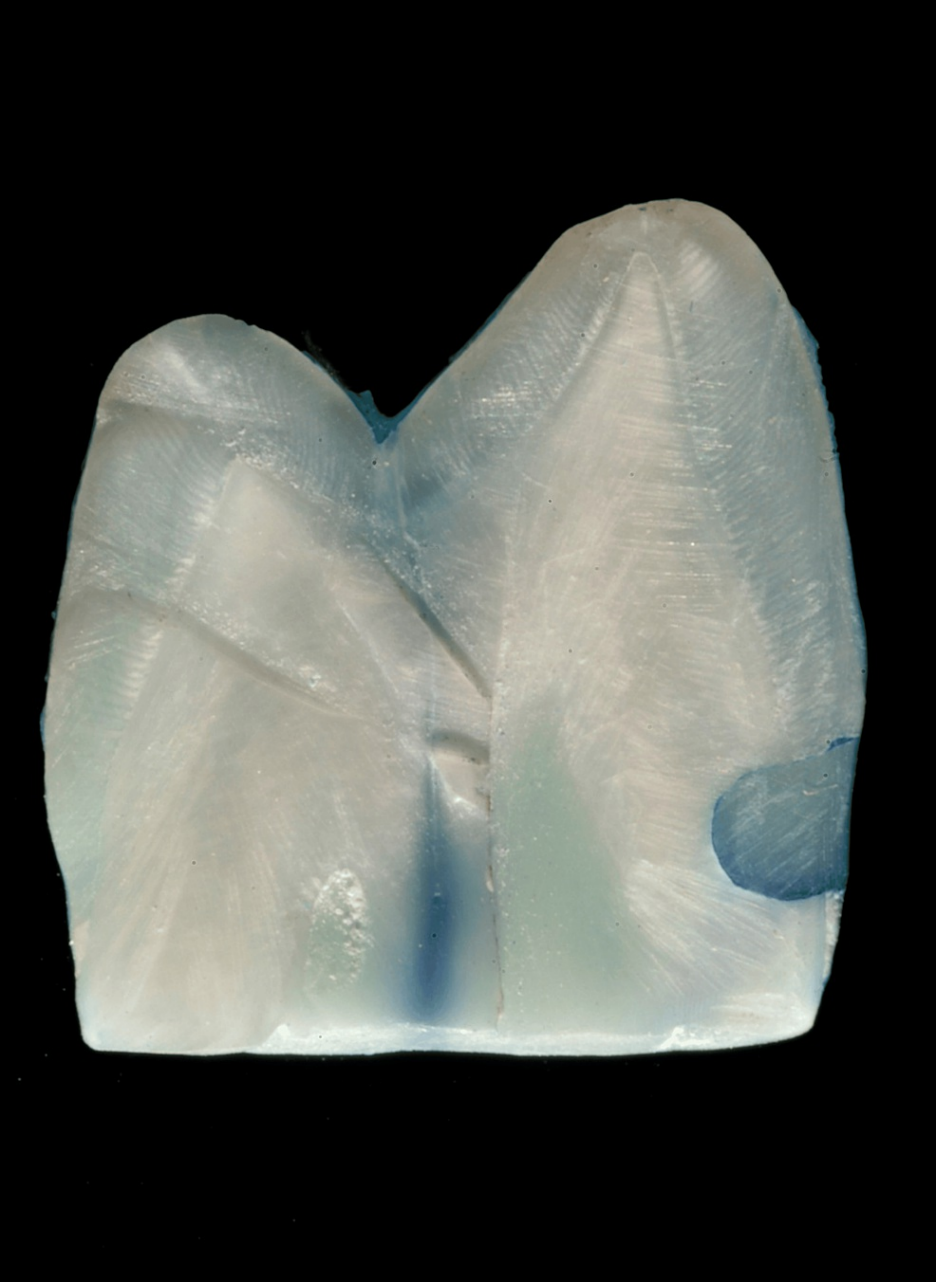
Stereomicroscopic image at 10x magnification showing dye penetration in Group A (total-etch adhesive system)

**Figure 2:**
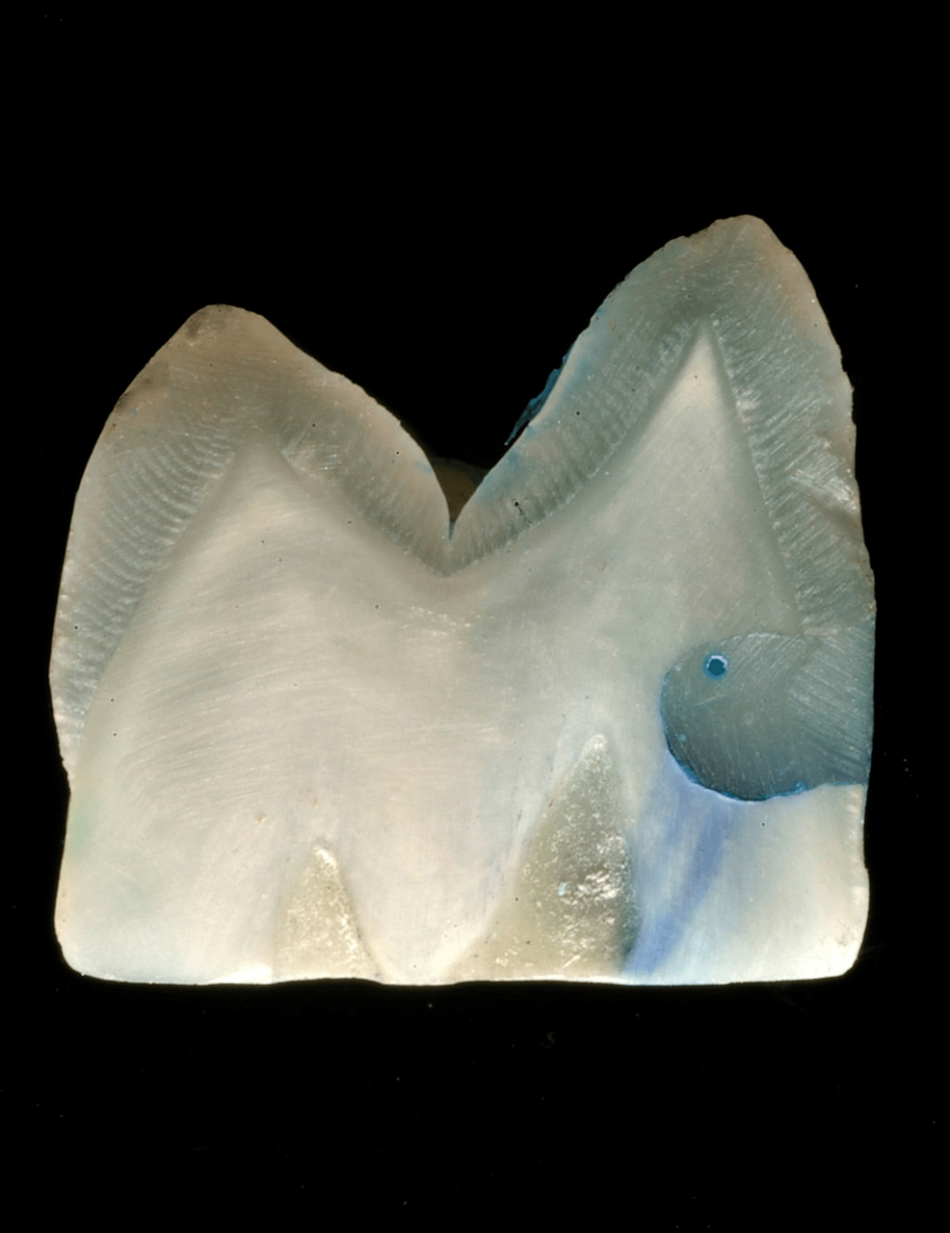
Stereomicroscopic image at 10x magnification showing dye penetration in Group B (self-etch universal adhesive system)

**Figure 3:**
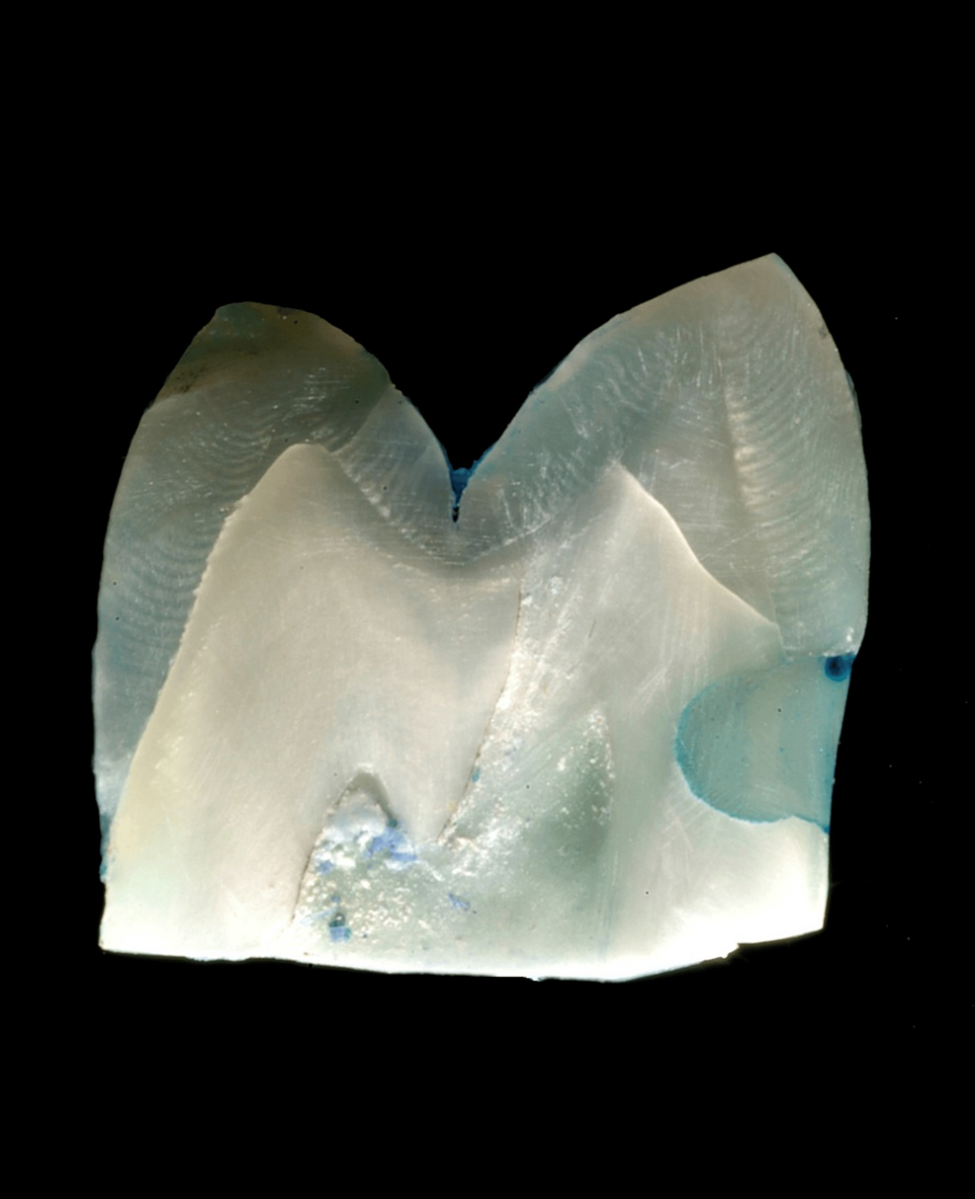
Stereomicroscopic image at 10x magnification showing dye penetration in Group C (nano-adhesive system)

The procedure later involved sectioning the radicular part horizontally 2.5 mm below the counter electrojet (CEJ), and then the longitudinal sectioning was done in a bucco-lingual direction at midline, with a low-speed diamond disc mounted in a mandrill. These sections were then visualised for dye penetration under a stereomicroscope at 10X magnification, and thus the images obtained were scored by an observer on the basis of scoring criteria (Figures 1-3, Table 1). An independent observer assessed the outcome of the experiment to promote transparency in the results.

**Table 1:** Scoring criteria Source: Ref. [3]

Score	Criteria
0	No evidence of dye penetration
1	Dye penetration along the interface to half the cavity depth
2	Penetration greater than half, not including axial wall
3	Penetration involving axial wall but not pulp
4	Penetration involving pulp

The collected data were entered in Microsoft Excel and subjected to statistical analysis using Statistical Package for Social Sciences (SPSS) (version 20.0; IBM SPSS Statistics for Windows, Armonk, NY). The level of significance was fixed at 5%, and p ≤ 0.05 was considered statistically significant. The Kolmogorov-Smirnov test and Shapiro-Wilks test were employed to test the normality of the data. Analysis of variance (ANOVA) and posthoc analysis were performed for the quantitative variables.

## Results

**Figure 4:**
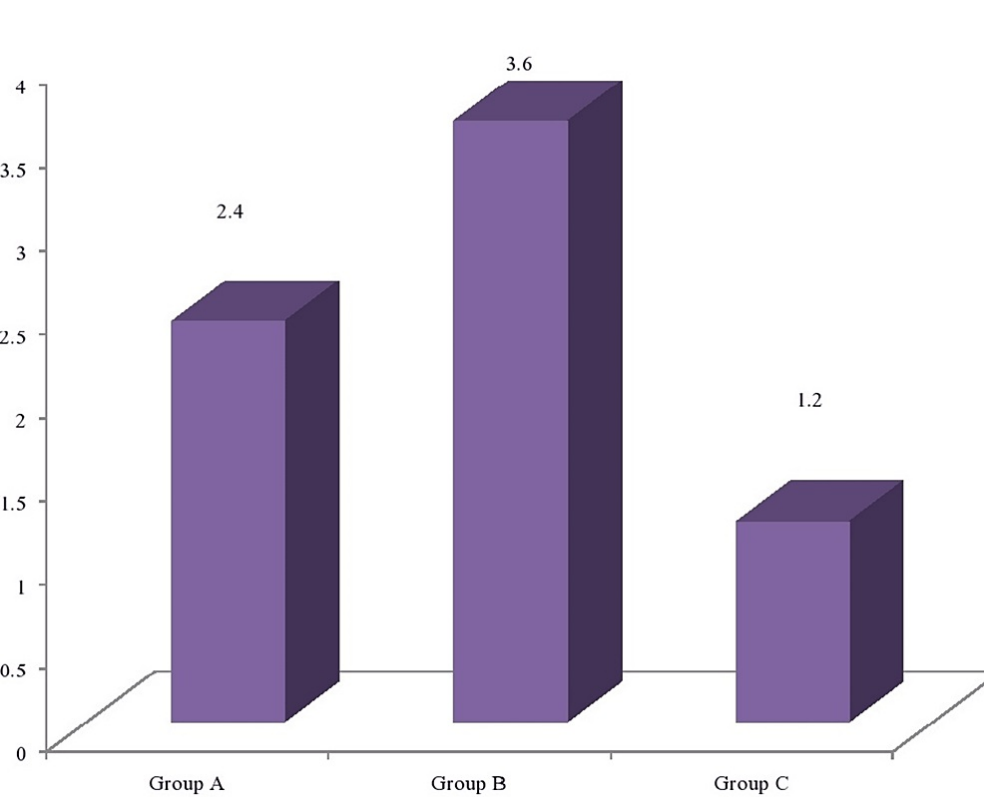
Comparison of the mean microleakage among the three groups

The present in-vitro study evaluated and compared the microleakage with total-etch, universal, and nano adhesive systems in Class V composite restorations. The selected 45 premolars were randomised into three groups and treated (i.e., Class V composite restorations) with total-etch (Tetric N-Bond) (Group A, n = 15), universal (Prime & Bond Universal) (Group B, n = 15), and nano adhesive (Futura Bond DC) (Group C, n = 15) (Figure 4). The mean microleakage score of Group B was found to be the highest (3.60 ± 0.55), followed by Group A (2.40 ± 0.55) and Group C (1.20 ± 0.45). A comparative evaluation revealed a significant difference (p value <0.001) between the three groups, with Group C having a significantly lower microleakage score as compared to both Group A and Group B (Table 2). Posthoc analysis revealed significant differences between all three groups assessed (Table 3).

**Table 2:** Mean microleakage score among the three groups

Group	n	Mean ± SD	F value	P value
Group A	15	2.40 ± 0.55	27.00	<0.001
Group B	15	3.60 ± 0.55		
Group C	15	1.20 ± 0.45		

**Table 3:** Comparison of the difference in the mean microleakage score between groups by the Tukey test

Comparison	Mean diff.	Mean diff. (%)	q value	P value	95% CI of diff.
Group A vs. Group B	-1.20	33.3	5.20	P < 0.01	-2.071 to -0.329
Group A vs. Group C	1.20	50.0	5.20	P < 0.01	0.329 to 2.071
Group B vs. Group C	2.40	66.7	10.39	P < 0.001	1.529 to 3.271

## Discussion

In the era of evolving biomedical sciences, and that too when restorative dentistry is emphasised, the bonding agents have evolved in a major manner, with advances being introduced every now and then. The factor that definitely triggered this evolution was the "need of the hour," be it from the patient’s perspective, such as aesthetics, or from the dentist’s perspective, such as longevity and "minimally invasive" or "minimum intervention" care [2].

Sealing is the most important property when a bonding agent is to be used. Adhesion (sealing) is inversely proportional to microleakage [2]. Various advancements have been made to improve the properties of adhesive systems, such as increasing the demineralizing potential of the etchant, adding fillers (nanofillers), and changing the resin matrix composition [9]. These developments are focused on reducing microleakage by strengthening the adhesion between the tooth surface and the restoration [10].

Due to the increased demand for aesthetic restorations and the availability of a variety of materials on the market, it is necessary to evaluate the efficacy of the new adhesive systems in reducing microleakage. The inability to maintain a marginal seal between restoration tooth interfaces has been found to be a primary reason for the failure of any restoration. In this study, maxillary and mandibular premolars have been chosen as the experiment’s samples as they are the most flexural teeth in the posterior region [11].

In the present study, Class V cavities were selected because restoring a cervical lesion with resin composites poses a challenge, particularly where no enamel is present for efficient bonding at the gingival margin. Dentin at the gingival margin shows higher organic components, tubular structure, fluid pressure, and lower surface energy that make bonding more difficult than enamel [12]. Further, the conventional Class V cavity employed in this study shows a high C-factor [13]. An increase in the C-factor could increase the shrinkage stress at the adhesive interface, thus impairing the sealing ability. This study used thermocycling to mimic intra-oral temperature variations and subject the restorations on the tooth to temperature extremes simulating the oral cavity.

The present study revealed that the nano-adhesive system, i.e., the eighth-generation dentin bonding agent (Futurabond DC), resulted in the least microleakage among the groups tested. The findings of this study are in accordance with the result of the study conducted by Somani et al. 9], in which eighth-generation nano-adhesives showed the least microleakage when compared with sixth- and seventh-generation bonding agents. The reason for the least microleakage of the nano-adhesive can be attributed to the fact that it contains polyfunctional adhesive monomers (phosphoric acid-modified methacrylate esters), which, when mixed with water, produce a favourable lower pH value of 1.4. The lower pH favours demineralization of the hydroxyapatite, which leads to enhanced infiltration of the monomers, forming an improved retentive pattern on the tooth surface. This results in better bonding and sealing ability, which might be the reason for reduced microleakage.

The higher microleakage of Group A (total-etch adhesive system) might be due to deeper demineralization produced by etching both enamel and dentin. Moreover, total-etch adhesive systems may be more susceptible to water degradation over time; this is because the polymerized primer of the "one bottle system" tends to be hydrophilic in nature. Our results are also in line with a study conducted by Shafigh et al. [14], in which they reported that an eighth-generation bonding agent had the highest bond strength (15.8 MPa) as compared to the fifth- and seventh-generation bonding agents (15.24 MPa and 11.24 MPa, respectively). High bond strength results in a better sealing ability of the adhesive, which might be the reason for reduced microleakage of the nano adhesive system (eighth generation) as compared to the total-etch adhesive (fifth generation).

The mean microleakage score of Group B (self-etch universal adhesive) was found to be higher than that of Group C (nano adhesive system). The reason could be that the self-etch universal adhesive has mild acids in its composition and does not demineralize enamel and dentin to the same extent as the nano-adhesive system (pH = 1.4). A similar result was reported by Kamble et al. [6] in their study, in which the highest mean tensile bond strength of 34.74431 MPa was observed in nano adhesives (Futurabond DC, Voco, Germany) compared to self-etch adhesives. They attributed the high bond strength of the nano adhesive system to the presence of nano-sized crosslinking filler particles (SiO2), leading to improved sealing ability and reduced microleakage as compared to self-etch universal adhesives.

Group B (universal adhesives used in self-etch mode) resulted in the highest microleakage score among the groups investigated. Self-etch universal adhesives, when applied to dentin and enamel, do not etch enamel to the same depth as 37% phosphoric acid (pH = 0.1-0.4), which is likely responsible for the high rates of microleakage in the enamel margins of cervical restoration due to their lower acidity (high pH) [15]. Yalniz et al. [16] compared the microleakage of Class V restorations with self-etch and selective-etch adhesive systems and reported that the two adhesive systems showed clinically acceptable microleakage values in two different application techniques.

The findings of this study summarize that nano-adhesives (eighth generation dentin bonding agents) showed the most promising result as it had the least microleakage at tooth-restoration interface, thus, providing superior sealing which may lead to increased longevity of the bonded restoration. The result of the present study is also in agreement with a study conducted by Kamath et al. [17], in which eighth-generation dentin bonding agents showed the least microleakage when compared to the sixth- and seventh-generation dentin bonding agents.

Strengths and limitations

The present study is one of the first to take into consideration thermocycling to stimulate temperature changes in the oral cavity. Additionally, the study evaluated microleakage, which is one of the major reasons for the failure of composite restorations. The utilisation of an eighth-generation nano adhesive further strengthens the findings of the study. The limited sample size and utilisation of the invitro study design, however, limit the findings of the present study and open new vistas of research in this field. Utilisation of advanced microleakage evaluation tools, such as microcomputed tomography, confocal laser scanning microscopes, and optical coherence tomography, may have further enhanced the evaluation in our study.

## Conclusions

Based on the findings of the present study, it was concluded that the nano adhesive system (eighth-generation bonding agent) presents better marginal integrity in comparison to the total-etch and universal adhesive systems. However, further studies with different study designs and a larger sample size should be performed in order to evaluate the clinical short- and long-term outcomes of newer adhesive systems.
